# Dialysis Disequilibrium Syndrome With Cerebral Edema in an Adult Patient Following the Initial Dialysis Session

**DOI:** 10.7759/cureus.67823

**Published:** 2024-08-26

**Authors:** Sahil M Patel, Varshini Venkatesan, Kenny J Murray

**Affiliations:** 1 Internal Medicine, Brookwood Baptist Health, Birmingham, USA

**Keywords:** chronic kidney disease (ckd), dialysis, diffuse cerebral edema, reverse urea effect, dialysis disequilibrium syndrome

## Abstract

Dialysis is a common treatment for removing toxins, electrolytes, and excess fluids due to impaired kidney function. A rare but life-threatening complication that can arise is dialysis disequilibrium syndrome (DDS) with cerebral edema. DDS is characterized by a range of neurological symptoms that may occur following dialysis. Its incidence is not well-established because it often presents with nonspecific symptoms, making diagnosis challenging. Here, we present a case of a 64-year-old female with a history of hypertension and chronic kidney disease stage 5, who sought evaluation for nausea and vomiting with coffee-ground emesis that began three weeks prior. Despite an initial blood transfusion stabilizing her hemoglobin with no further hematemesis, she developed DDS with cerebral edema after her first dialysis session. The condition was managed with 3% hypertonic saline, which quickly resolved both her cerebral edema and neurological symptoms. She tolerated subsequent dialysis sessions without complications and was discharged with a follow-up arranged with nephrology and an outpatient dialysis chair. This case report reviews the clinical features, risk factors, pathophysiology, management, and treatment goals for DDS. In patients commencing dialysis, particular attention should be given to preventing DDS, especially in those with elevated blood urea nitrogen levels above 100 mg/dL. Prompt recognition and treatment are crucial to balance the osmotic gradient and prevent severe outcomes, such as cerebral edema and death.

## Introduction

Dialysis is a widely utilized treatment for filtering out toxins, electrolytes, and excess fluid resulting from impaired kidney function. It is commonly administered to patients with end-stage renal disease (ESRD), acute kidney injury, or those requiring the removal of dangerously high toxin levels. A rare but life-threatening complication associated with dialysis is dialysis disequilibrium syndrome (DDS) with cerebral edema [1]. DDS is characterized by a range of neurological symptoms that may arise after dialysis [2]. The condition was first reported by Kennedy et al. in 1962 [3]. The exact incidence of DDS remains unclear due to its often nonspecific symptoms, such as headache, nausea, blurred vision, disorientation, and dizziness, which complicate diagnosis [4]. The incidence may have decreased with advancements in dialysis techniques and dialysate options [4]. This report presents a case of a patient with ESRD who developed DDS with cerebral edema following an initial dialysis session.

## Case presentation

A 64-year-old woman with a history of hypertension, chronic kidney disease (CKD) stage 5 (not on dialysis), and gastroesophageal reflux disease presented to the emergency department for evaluation of nausea and vomiting with coffee-ground emesis that had started three weeks prior. She reported diffuse abdominal pain but denied hematochezia or melena. She also denied any use of pain medication or alcohol.

**Figure 1:**
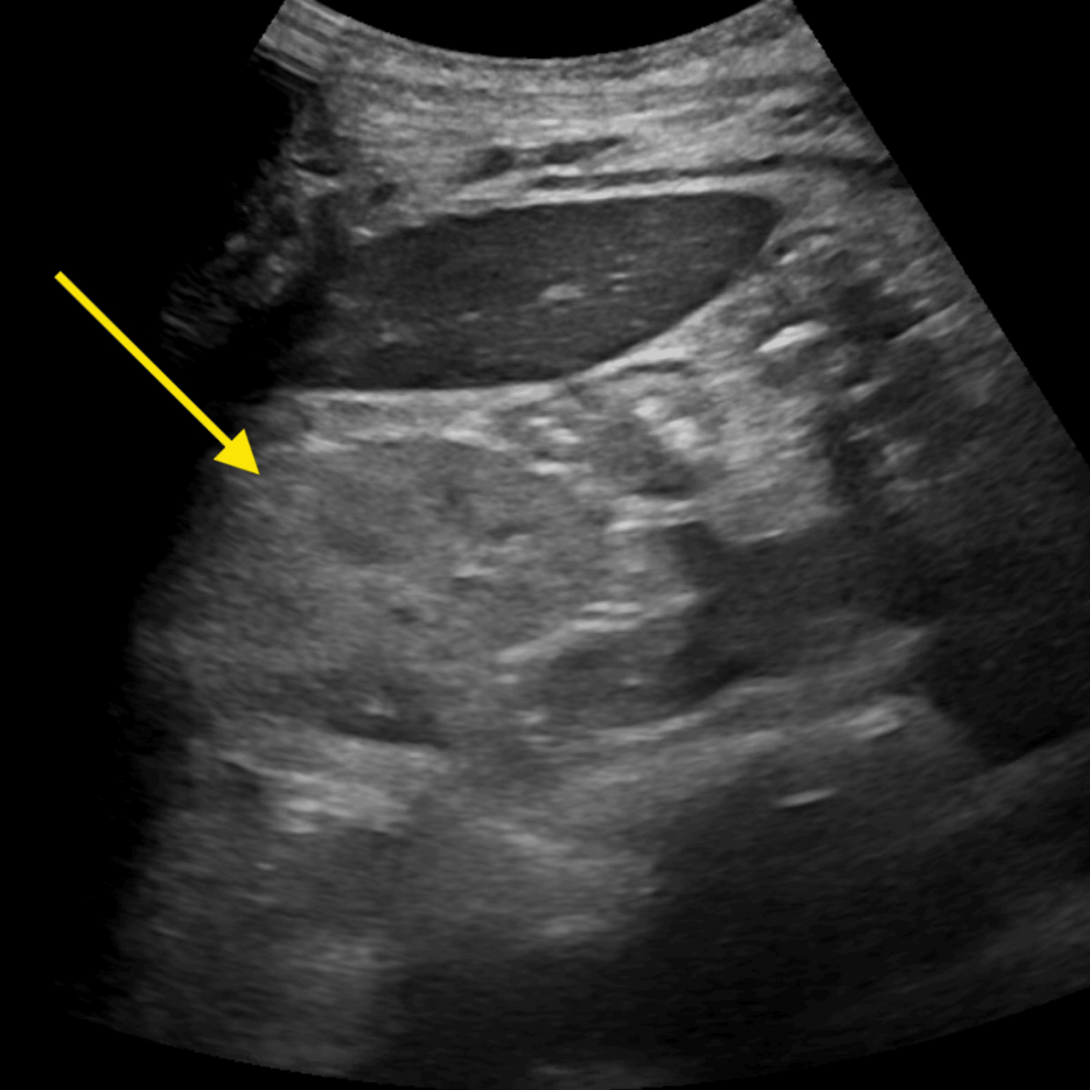
Abdominal ultrasound The abdominal ultrasound revealed a hyperechoic right kidney.

**Figure 2:**
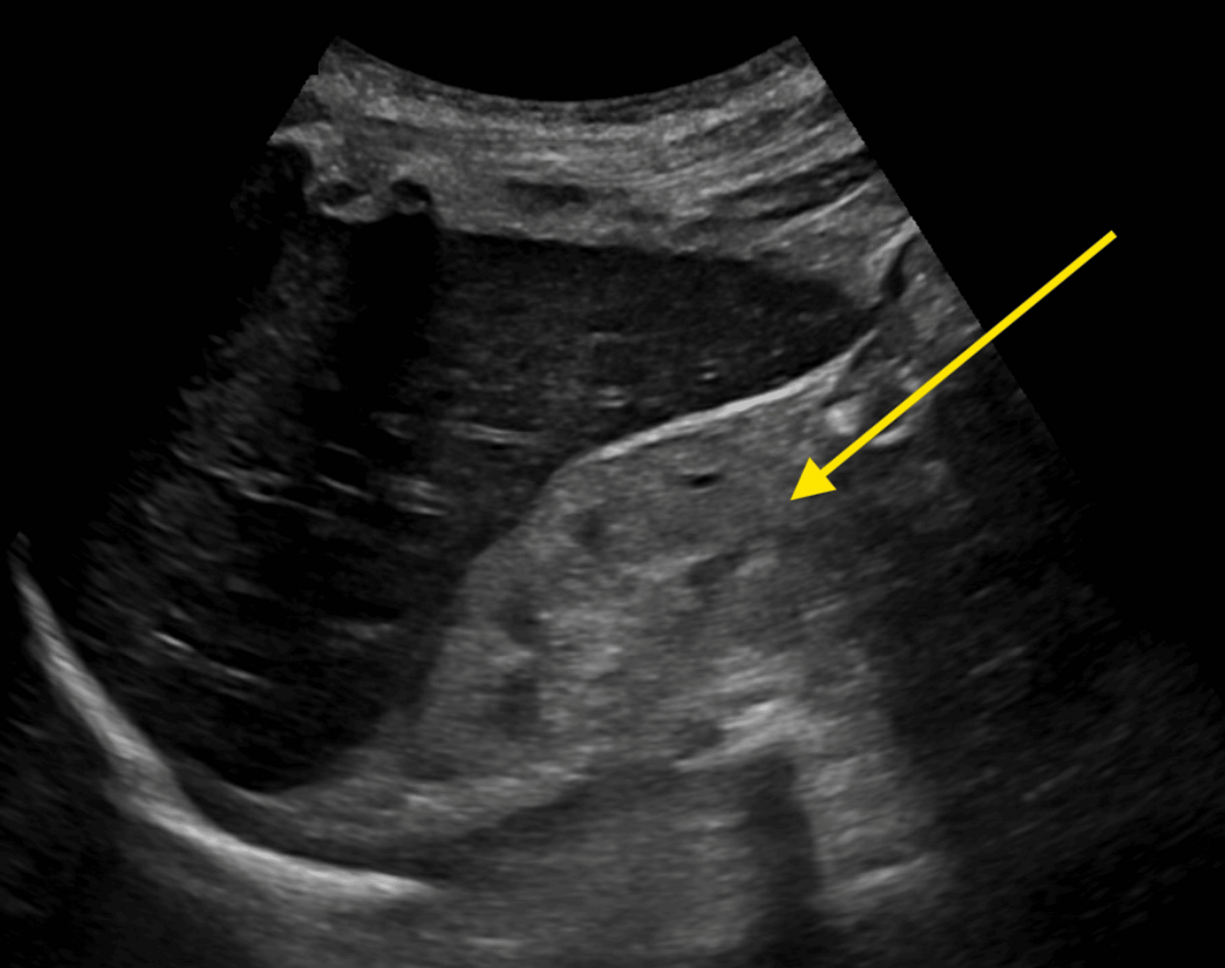
Retroperitoneal kidney ultrasound The retroperitoneal kidney ultrasound showed no signs of hydronephrosis.

Vital signs revealed tachycardia at 128 beats per minute and tachypnea at 26 respirations per minute. A physical examination showed a thin, disheveled woman in moderate distress with diffuse abdominal tenderness. She was alert and oriented to person, place, and time. Initial lab work indicated a hemoglobin level of 4.4 g/dL, hematocrit of 14.2%, sodium 141 mEq/L, potassium 6.2 mEq/L, bicarbonate 3 mEq/L, anion gap 22 mEq/L, blood urea nitrogen (BUN) 164 mg/dL, creatinine 29.30 mg/dL, and phosphorus 12.6 mg/dL (Table 1). The urine drug screen was unremarkable. A CT of the abdomen and pelvis with contrast, as well as a chest X-ray, showed no significant abnormalities. Abdominal ultrasound revealed a hyperechoic right kidney, indicative of CKD (Figure 1), while the left kidney was not visualized. Retroperitoneal kidney ultrasound showed no signs of hydronephrosis (Figure 2).

**Table 1:** Laboratory results AG, anion gap; BUN, blood urea nitrogen; Cr, creatinine; HCO_3_, bicarbonate; HCT, hematocrit; Hgb, hemoglobin; K, potassium; Na, sodium; Phos, phosphorus

Lab test (reference range)	Initial lab work before dialysis	Repeat lab work after the onset of encephalopathy
Hgb (11.5-15.2 g/dL)	4.4 g/dL	8.4 g/dL
Hct (33-45%)	14.20%	24.10%
Na (134-144 mEq/L)	141 mEq/L	140 mEq/L
K (3.5-5.1 mEq/L)	6.2 mEq/L	3.9 mEq/L
HCO3 (21-32 mEq/L)	3 mEq/L	19 mEq/L
AG (2-12 mEq/L)	22 mEq/L	20 mEq/L
BUN (7-18 mg/dL)	164 mg/dL	47 mg/dL
Cr (0.55-1.02 mg/dL)	29.30 mg/dL	9.11 mg/dL
Phos (2.5-4.9 mg/dL)	12.6 mg/dL	4.2 mg/dL

Gastroenterology was consulted due to concerns about a gastrointestinal bleed. The patient received three units of packed red blood cells, resulting in an appropriate increase in hemoglobin to 8.4 g/dL and hematocrit to 24.1% (Table 1), which remained stable throughout her hospitalization. Nephrology was also consulted to evaluate the patient’s CKD stage 5 and assess for potential progression to ESRD. IV sodium bicarbonate was initiated, and the patient underwent emergent dialysis.

**Figure 3:**
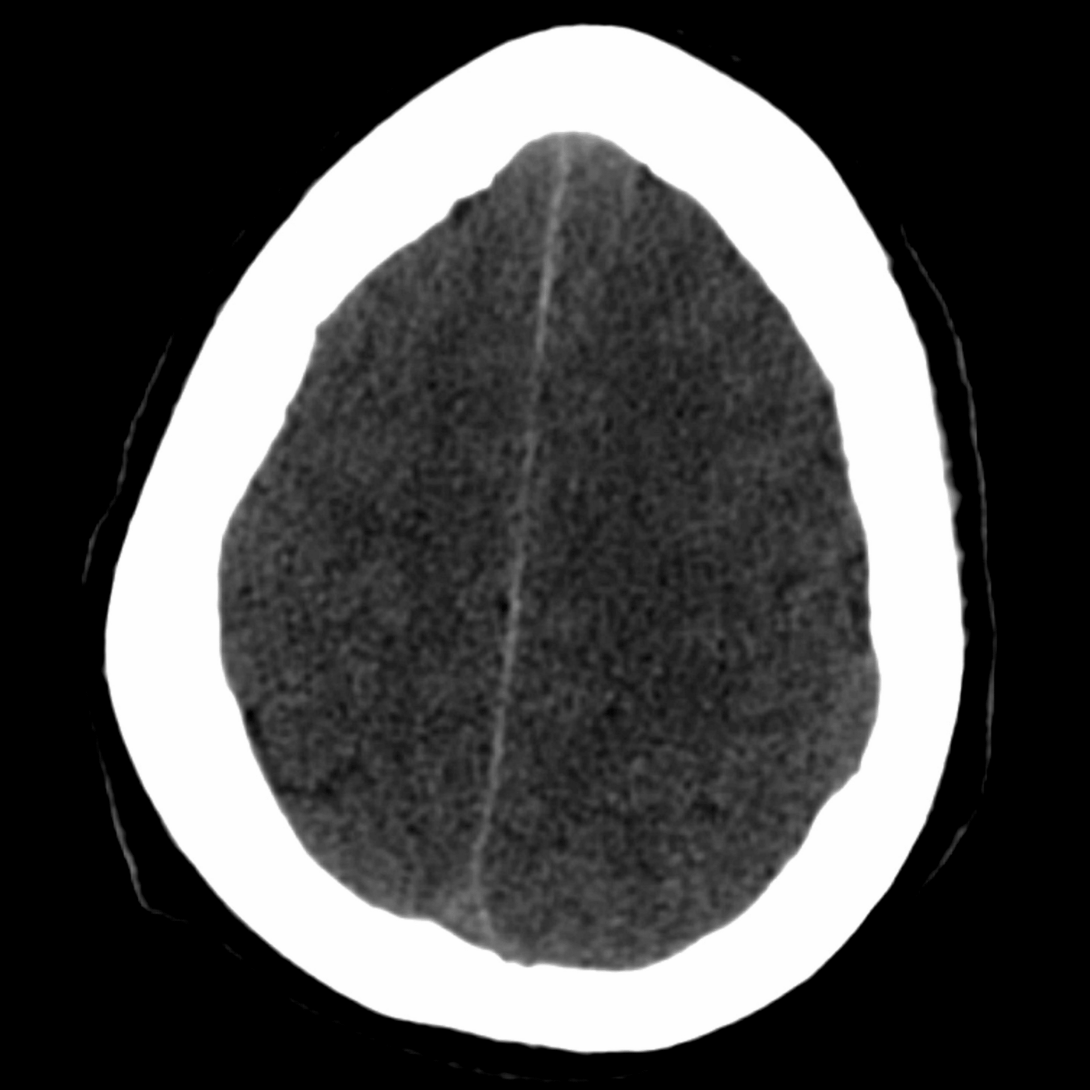
CT scan of the brain CT of the brain shows diffuse loss of CSF spaces along the cerebral convexities and blurring of the gray-white matter junction, indicating cerebral edema.

Shortly after her four-hour hemodialysis session, the patient developed encephalopathy, becoming unresponsive and disoriented. Point-of-care glucose testing ruled out hypoglycemia, and no seizure activity was noted on physical examination. A stat CT brain scan (Figure 3) revealed diffuse loss of CSF spaces along the cerebral convexities with blurring of the gray-white matter junction, indicating cerebral edema. No intracranial hemorrhage or significant mass effect was observed. CT angiography of the head and neck showed no flow-limiting stenosis. Neurology was consulted, and an EEG showed no evidence of seizure activity. Repeat laboratory tests showed sodium 140 mEq/L, potassium 3.9 mEq/L, bicarbonate 19 mEq/L, anion gap 20 mEq/L, BUN 47 mg/dL, creatinine 9.11 mg/dL, and phosphorus 4.2 mg/dL (Table 1).

After consultations with neurology and nephrology, the diagnosis of DDS with cerebral edema was confirmed. The patient was treated with 250 mL of 3% hypertonic saline, administered at a rate of 50 mL/hr. A repeat CT brain scan performed five hours later showed improvement, with no definitive intracranial abnormalities. The following day, the patient’s neurological examination showed significant improvement, and her mentation returned to baseline. After the resolution of DDS, she successfully underwent additional dialysis without complications. A permanent dialysis catheter was placed, and the patient was discharged with nephrology follow-up and a schedule for regular hemodialysis.

## Discussion

DDS has a wide variety of clinical manifestations, ranging from milder symptoms such as headaches, nausea, dizziness, or cramps to more severe symptoms such as seizures or altered mental status [1]. Our patient presented with the more severe symptom of altered mental status. Seizures were ruled out with an unremarkable EEG. There are several risk factors that increase the risk of developing DDS, such as: undergoing an initial dialysis session or sudden change in dialysis regimen; being a child or elderly; having a BUN greater than 175 mg/dL; or having preexisting neurological diseases that cause cerebral edema or increase the blood-brain barrier (BBB) permeability [2,4,5]. Our patient had several risk factors, as this was her initial dialysis session, she was elderly, and she had an elevated BUN, although not as high as 175 mg/dL.

The pathophysiology behind DDS is not well defined, but there are three major proposed theories. The first is the reverse urea effect. The theory here is that patients who have chronically elevated BUN levels will also have a correspondingly high urea concentration in the CSF and brain cells [6]. Urea is transported across the BBB via specific urea transporters (UT-B1), which have a slower diffusion rate than water, which crosses via aquaporin-4 channels [1]. As a result, during dialysis, plasma concentrations of urea decrease rapidly, faster than the rate at which it can be transported across the BBB. This creates an osmotic gradient that pulls water across the BBB into the CSF, leading to cerebral edema with increased intracranial and intraocular pressures.

The second theory involves intracerebral acidosis. Although less evident, the thought process here is that there is a decreased intracellular pH in brain cells due to the inability of the kidneys to excrete hydrogen ions [1]. The decreased pH causes protein-bound cations such as potassium and sodium to dissociate and become osmotically active [7]. In addition, the presence of metabolic acidosis necessitates compensatory respiratory hyperventilation or exogenous bicarbonate administration. When bicarb is rapidly administered, the sudden shift in pH suppresses the compensatory respiratory hyperventilation, which leads to an increase in pCO_2_ [1]. This increased CO_2_ can diffuse rapidly across the BBB, which further acidifies the CSF.

The third theory involves local inflammation and cerebral edema. The relationship between cerebral ischemia and hypotension during dialysis is not well established, but studies have shown that cerebral ischemia was present in 23.5% of hemodialysis sessions, with only 31.9% of those being symptomatic [8]. In addition, a 10 mmHg drop in mean arterial pressure has a strong association with an increase in the risk of ischemic events [8]. This can lead to the development of cytotoxic edema through the depletion of adenosine triphosphate and the intracellular shift of sodium and water [1]. Our patient’s experience would be most consistent with the first two theories, as her BUN rapidly dropped from 164 mg/dL to 47 mg/dL and she received IV sodium bicarbonate, which rapidly increased her bicarbonate from 3 mEq/L to 19 mEq/L. Both of these factors likely played a contributing role to the development of the cerebral edema, which was seen on her initial CT scan.

Most of the management of DDS is geared toward prevention rather than treatment. Several modalities exist to prevent DDS, including the administration of osmotically active substances, increasing sodium dialysate levels, slow and gentle initial dialysis, and restricting the clearance of urea to prevent the creation of an osmotic gradient [2]. For patients who are receiving their initial dialysis session, shorter two-hour sessions at lower blood and dialysate flow rates can be done, followed by consecutive daily sessions, which can be slowly up-titrated to match outpatient settings [9]. Special considerations should be given to patients being initiated on dialysis with a BUN greater than 100 mg/dL or those with neurological symptoms at initial presentation [2].

Once symptoms have developed, the aim is to reduce the osmotic gradient across the BBB, which is achieved by increasing the concentration of osmotically active solutes. The first step is to initiate sodium remodeling by altering the sodium dialysate bath [10]. Symptom resolution is expected within 30 minutes, and as a result, dialysis does not need to be stopped [2]. However, if symptoms do persist, then dialysis should be stopped and other underlying causes should be explored [2]. In patients who present with severe symptoms of DDS, especially those whose symptoms persist despite sodium remodeling, efforts should be taken to decrease intracerebral pressure by increasing osmotically active particles in the serum [2]. Data is limited regarding the precise method to accomplish this, whether it be through hypertonic saline, mannitol, or other methods. Given that this was our patient’s initial dialysis, we should have taken extra caution in trying to prevent DDS from developing. When she did develop symptoms, it was after her dialysis session had been completed, so there was no opportunity for sodium remodeling. She was given 3% hypertonic saline, which successfully decreased the cerebral edema, which was seen on her initial CT brain, and resolved all of her neurological symptoms.

## Conclusions

When initiating dialysis, special attention must be given to preventing the development of DDS, particularly in patients with a BUN greater than 100 mg/dL. If symptoms of DDS arise, the goal is to increase osmotically active particles in the serum to counteract the osmotic gradient across the BBB. DDS is usually reversible with prompt recognition and appropriate intervention. However, failure to identify and address DDS can lead to cerebral edema and, potentially, death.
